# Charge transport mechanism in lead oxide revealed by CELIV technique

**DOI:** 10.1038/srep33359

**Published:** 2016-09-15

**Authors:** O. Semeniuk, G. Juska, J.-O. Oelerich, M. Wiemer, S. D. Baranovskii, A. Reznik

**Affiliations:** 1 Chemistry and Material Science Program, Lakehead University, 955 Oliver Road, Thunder Bay, ON, P7B 5E1, Canada; 2 Department of Solid State Electronics, Vilnius University, Saulėtekio 9 III k., 10222 Vilnius, Lithuania; 3 Faculty of Physics, Philipps University Marburg, Renthof 6, 35032 Marburg, Germany; 4 Department of Physics, Lakehead University, 955 Oliver Road, Thunder Bay, ON, P7B 5E1, Canada.; 5 Advanced detection devices department, Thunder Bay Regional Research Institute, 290 Munro Street, Thunder Bay, ON, P7A 7T1, Canada.

## Abstract

Although polycrystalline lead oxide (PbO) belongs to the most promising photoconductors for optoelectronic and large area detectors applications, the charge transport mechanism in this material still remains unclear. Combining the conventional time-of-flight and the photo-generated charge extraction by linear increasing voltage (photo-CELIV) techniques, we investigate the transport of holes which are shown to be the faster carriers in poly-PbO. Experimentally measured temperature and electric field dependences of the hole mobility suggest a highly dispersive transport. In order to analyze the transport features quantitatively, the theory of the photo-CELIV is extended to account for the dispersive nature of charge transport. While in other materials with dispersive transport the amount of dispersion usually depends on temperature, this is not the case in poly-PbO, which evidences that dispersive transport is caused by the spatial inhomogeneity of the material and not by the energy disorder.

Non-crystalline materials are of great significance to a variety of applications since they offer a low cost reliable technology combined with reproducible outcomes. This is, of course, true if these materials achieve the desirable performance. Therefore thorough study of material properties as an input to technology optimizations is crucially needed and frequently opens new technological horizons. One example is a-Si:H: by revealing a way to passivate dangling bonds in a-Si with hydrogen, it opened a new era in large area electronics based on a-Si:H. Another example is amorphous selenium (a-Se): inventing ways to stabilize a-Se against crystallization and improve charge transport gave rise to a broad range of applications; from the first Xerox photocopiers, to Vidicon pick-up tubes, and finally to the ultra-sensitive High-gain Avalanche Rushing (HARP) TV cameras^1^. A natural transition to thick a-Se layers for adequate X-ray absorption resulted in a new generation of high-resolution direct conversion mammography detectors with a-Se layer as the X-ray-to-charge transducer^2^. a-Se based detectors revolutionized X-ray medical imaging, offering diagnostic capabilities in the mammographic energy range (around 20 keV), which was not achievable by other commercial mammographic detectors^3^.

Besides a-Se, there have been a number of other non-crystalline photoconductors, which have been investigated for use as X-ray-to-charge convertors in X-ray detectors^4^,^5^,^6^,^7^,^8^,^9^,^10^,^11^,^12^,^13^,^14^,^15^,^16^,^17^,^18^, although they are not yet mature enough for commercial use^19^. Polycrystalline Lead Oxide (PbO) holds a special place in this list since, similar to a-Se, it has been used previously in optical imaging, in so-called Plumbicon video pick-up tubes. Due to the good photoconductive properties of PbO, Plumbicons were used extensively in broadcast, fluoroscopy and digital subtraction angiography in conjunction with an image intensifier. The wide band gap of PbO in combination with the high X-ray absorption, low electron-hole pair creation energy^17^,^18^, and the absence of K absorption edges up to 88 keV X-rays^19^ makes polycrystalline PbO a good candidate to expand the advantages of the direct conversion X-ray detection scheme over the fluoroscopic (around 70 keV) energy range due to its much higher atomic number than in a-Se.

In 2005, Simon *et al*. evaluated the imaging performance of the first prototype of a direct conversion flat panel imager with a thick (∼300 μm) layer of poly-PbO^18^, which showed high spatial resolution with an effective fill factor close to unity. However, it did not show the expected high conversion efficiency and adequate temporal behavior for fluoroscopic applications. Thus, optimization of PbO technology is required to make “detector grade” thick layers. This, in turn, needs a comprehensive analysis of the electronic characteristics of the material in order to understand the interplay between X-ray performance and material properties.

Despite continuous interest in polycrystalline PbO since 1960’s, the mechanisms of charge carrier transport and recombination – two major features to be used as a feedback for technology optimization, have not yet been clarified. The data on charge carrier mobility has mostly been obtained from single crystal materials. For example, Keezer *et al*. measured electron mobility, *μ*_*e*_, and mobility-lifetime product, *μ*_*e*_*τ*_*e*_, (where *τ*_*e*_ is the electron lifetime) of single crystals produced by the hydrothermal technique. The corresponding values were reported to be: *μ*_*e*_ = 100 cm^2^/Vs, *μ*_*e*_*τ*_*e*_ = 10 cm^2^/V for tetragonal and *μ*_*e*_ = 50 cm^2^/Vs, *μ*_*e*_*τ*_*e*_ = 4 × 10^−4^ cm^2^/V for orthorhombic single crystal PbO^20^. At the same time, Broek reported *μτ* = 2 × 10^−9^ cm^2^/V for a tetragonal PbO crystal, though the type of carrier was not specified^21^. For polycrystalline material, the reported data are on the mobility-lifetime product only. Schottmiller measured *μτ* to be ∼10^−7^ cm^2^/V and ∼10^−9^ cm^2^/V for tetragonal and orthorhombic phases of poly-PbO, respectively (the type of charge carrier was not specified)^22^. Simon *et al*. reported *μτ* = 4.4 × 10^−7^ cm^2^/V for evaporated layers containing fractions of both phases^18^. Although the type of charge carrier in ref. 18 was not specified, the experimental results presented there were later treated by Kabir, allowing him to derive the mobility-lifetime products for electrons and holes, *μ*_*e*_*τ*_*e*_ = 3.5 × 10^−7^ cm^2^/V and *μ*_*h*_*τ*_*h*_ ≈ 10^−8^ cm^2^/V, respectively^23^. The wide scatter of the reported values is not surprising since the lifetime *τ* depends on the carrier concentration, which makes evaluation of this parameter very sensitive to the measurement technique used. In contrast to the mobility-lifetime product, the carrier drift mobility *μ* is an objective parameter to evaluate transport in any semiconductor. However, no direct measurements of carrier mobility in polycrystalline PbO have been reported yet.

In the current manuscript, we provide a comprehensive analysis of the temperature and field dependencies of the mobility of the most mobile charge carriers (holes) in polycrystalline PbO using the conventional time-of-flight (TOF)^24^ and photo- charge extraction by linear increasing voltage (photo-CELIV)^25^ techniques. It appears that holes in poly-PbO conduct electrical current in a dispersive regime, in which the carrier mobility is time-dependent and therefore it does not have a universal value that could be treated as a characteristic one for the material. In order to apply the CELIV technique in the case of the dispersive regime, it was necessary to extend the CELIV theory to account for the dispersive nature of charge transport.

## Results

### Experimental data on charge transport by TOF

**Figure 1:**
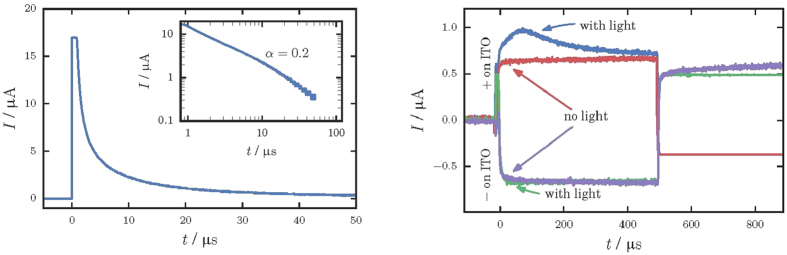
(**a**) TOF current transient measured at *F* = 1 V/μm is plotted in linear scale. The inset shows the current transient in double logarithmic scale. The TOF measurements were performed with excitation wavelength of 355 nm. (**b**) CELIV current transients with and without illumination for different polarities of applied voltage. Note: for both TOF and CELIV measurements an excitation wavelength of 355 nm was used for near-surface photogeneration.

The typical TOF current transient for holes in the polycrystalline PbO is shown in Fig. 1a. A featureless drop in the photocurrent indicates that transport occurs in the so-called dispersive mode, for which the carrier mobility decreases in the course of time. Generally, the time dependencies of the photocurrent (Δ*i*) for the dispersive transport mode are given by the following expression^26^:


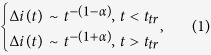


where *t*_*tr*_ is the transit time of the carriers through the sample and *α* – is the so-called dispersion parameter. The values of *α* usually vary for different materials between zero (the extreme case of dispersion) and unity (non-dispersive transport). In order to determine *α*, we plot in the inset to Fig. 1a the current transient in the double logarithmic scale: *α* = 0.2 is found from the initial slope of the signal indicating a very high degree of dispersion for transport of holes in PbO. It is, however, not possible to check the *α* value from the current decay at *t* > *t*_*tr*_ since the exact inflection point and the transit time *t*_*tr*_ is hard to determine even in the double logarithmic plot. Therefore we have chosen the photo-CELIV technique as an alternative to the TOF in order to check the value of *α*. The theoretical support of the photo-CELIV technique has been developed so far only for the case of a non-dispersive charge transport^25^. In the next section, we present our experimental results obtained by the photo-CELIV technique and in the subsequent section we develop a theory for the photo-CELIV in the case of dispersive transport.

### Experimental data on charge transport by photo-CELIV

The typical photo-CELIV signal measured with a strongly absorbed 355 nm light pulse is shown in Fig. 1b. When positive polarity is applied to the illuminated electrode, a clear photo-peak is seen on the CELIV trace and a photocurrent is attributed to the drift of photogenerated holes. The photocurrent rises rapidly until the majority of mobile carriers have been extracted at *t*_*peak*_, and then drops precipitously. Please, note that the splash of a signal of reverse polarity after the carrier extraction is due to the shape of the applied voltage pulse, which consists of a positive voltage ramp (needed for carrier extraction) followed by symmetrical negative ramp that drives the signal below the initial value.

When negative polarity is applied to the same illuminated electrode, only minor deviation from the capacitive signal with no pronounced photo-peak is observed, suggesting that the duration of the experiment was insufficient to observe extraction of slow electrons at the applied electric fields. Previously, it was shown that photo-CELIV with uniform volume absorption provides the most accurate measurements of the mobility^27^. Therefore, strongly absorbed light (355 nm) was used to determine the type of carriers, while a wavelength of 532 nm was used for mobility measurements.

### Analysis of photo-CELIV for dispersive transport

If charge carriers are generated in the entire volume of a sample, the total current is given by^25^:


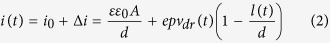


where *i*_0_ is the capacitive component of the total current (equation (2) is applicable when Δ*i* < *i*_0_), *εε*_0_ is the dielectric permittivity of the material, *e* is the elementary charge, *p* is the concentration of carriers, *v*_*dr*_ is the drift velocity, *A* = *dU*/*dt* is the slope of the voltage ramp and *l*(*t*) is the extraction depth that equals to:


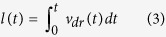


In the case of dispersive transport, the motion of the drifting packet of the photo-generated charge slows down with time, resulting in the time dependent drift mobility:


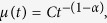


where *C* is the time-independent coefficient. For a voltage that is linearly increasing with time, drift velocity is given by:


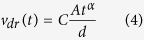


According to equations (3) and (4), the extraction depth becomes dependent on *α* as:


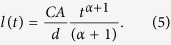


Concomitantly, the time needed to reach the peak of the photocurrent, *t*_*peak*_, also becomes dependent on *α*. In order to find out this dependence, we substitute equations (4) and (5) into equation (2) and determine *t*_*peak*_ by differentiating equation (2) over time and equating the result to zero:


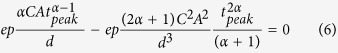


or:


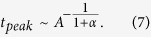


The carrier mobility is related to the peak position as:


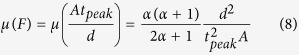


In the case of a non-dispersive transport equation (8) is transformed into 
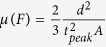
 in full agreement with the previously suggested expression^25^. Equation (8) allowed us to calculate *μ*_*h*_(*F*) using the measured dependence in the technically achievable range of electric fields (limited by the system noise and the bandwidth of the signal generator).

**Figure 2:**
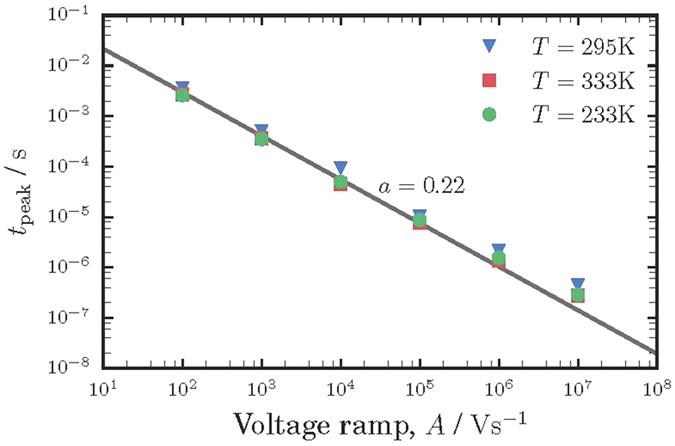
Dependence of *t*_*peak*_ on voltage ramp *A* for different temperatures. When plotted on a log-log scale, *t*_*peak*_ shows linear dependence on *A*, with a slope, corresponding to *α* = 0.22. Note: uniformly absorbed light of 532 nm was used for *t*_*peak*_ measurements.

**Figure 3:**
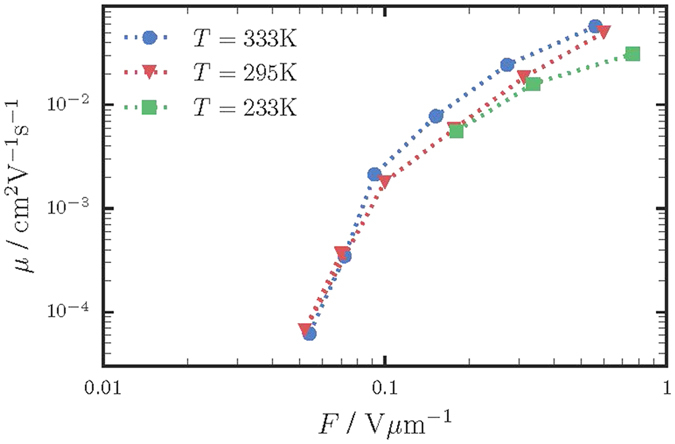
Dependence of hole mobility on electric field for different temperatures. Hole mobility tends to reach ∼0.1 cm^2^/Vs at *F* ∼ 1 V/μm, while remaining almost temperature independent.

In Fig. 2, *t*_*peak*_ is plotted as a function of *A* for different temperatures between 233 K and 333 K. In accordance with equation (7), the slope of log(*t*_*peak*_) *vs* log(*A*) curve yields the dispersion parameter *α* which is found to be 0.22. This is in perfect agreement with the value *α* = 0.2 determined above from the slope of the TOF signal. Remarkably, the slope in the *t*_*peak*_(*A*) dependence shown in Fig. 2 is almost insensitive to the change in temperature, which evidently shows that *α* is temperature-independent. As shown in Fig. 3, mobility also does not demonstrate any significant temperature dependence.

Numerous recent studies suggested that photo-CELIV and CELIV mobility calculations using equations are not necessarily reliable unless combined with numerical simulations^28^,^29^,^30^,^31^. In our work we do not implement numerical simulations so the question might arise on whether our theoretical analysis is sufficiently accurate. It should be noted that numerical simulations have been proven unavoidable when specific experimental conditions are not accounted for when using traditional CELIV equations. Such conditions are: (i) charge carriers are generated close to the surface by strongly absorbed light. This results in non-uniform charge carrier distribution through the sample; (ii) the photocurrent (Δ*i*) exceeds the *i*_0_, which is a capacitive component of the total current *i*, thus redistributing the internal electric fields; (iii) the linearly increasing voltage is comparable to the built-in barrier voltage on the electrode/semiconductor interface. In this case, a fraction of applied voltage is used to overcome the built-in potential. An offset voltage must be then used to compensate for this phenomenon, otherwise incorrect mobility values would be obtained.

In our study, the charge carriers were generated by the uniform volume absorption, which was shown to provide the most accurate measurements of the mobility^27^. At the same time, the light intensity was carefully adjusted in such a way that the magnitude of the photocurrent Δ*i* did not exceed the magnitude of the capacitive signal *i*_0_ (Fig. 1b), thus excluding the internal field modulation^25^. Figure 1b also shows that only capacitive signal is observed when sample is not irradiated with light, indicating that the equilibrium carriers do not affect the photo peak measurements^25^. The total applied voltage to the sample was about 20 V, which is significantly larger than the built-in barrier potential that is usually of the order of a volt. Therefore only a minimal effect (if any) of the barrier is expected on the measured results.

By comparison with the results of numerical simulations, Lorrmann *et al*.^28^ have developed a criterion for the validity of the simplified approach based on equations, as derived in the pioneering papers for the CELIV technique. According to this criterion, equations without numerical simulations are reliable if the dimensionless voltage slope *A*′ is larger than unity:


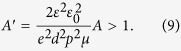


Although criterion (9) has been suggested only for the case of equilibrium carriers^28^, one might try to use it for the case of photo-CELIV just for estimations. In order to make the estimates, one should take into account that different mobility values shown in Fig. 3 were measured at different *A* values shown in Fig. 2. For all our experiments, the maximum voltage bias has been kept constant *U* = 10 V, while voltage pulse time was varied, providing different voltage ramps *A*. Therefore, for each mobility measurement its own *A* has been set. For instance, *μ* = 6 × 10^−2^ cm^2^/Vs was measured at *A* = 10^7^ V/s, while *μ* = 2 × 10^−2^ cm^2^/Vs corresponds to *A* = 10^6^ V/s, etc. Inserting these (*A*, *μ*) values along with *d* = 5 μm, *p* = 5 × 10^19^ m^−3^, *ε* = 13 into criterion (9) one can claim, that the criterion is fulfilled for *A* values between 10^4^ and 10^7^ V/s. For two lowest *A* values of 10^2^ V/s and 10^3^ V/s, the criterion (9) would be valid for *μ* smaller than 1.65 × 10^−5^ cm^2^/Vs and 1.65 × 10^−4^ cm^2^/Vs, respectively. As it is seen from Fig. 3, the corresponding measured mobilities are slightly larger than these values, and one can only claim the fulfilling of criterion (9) by order of magnitude. More theoretical study is necessary to develop a similar criterion for the case of non-equilibrium carriers relevant to the photo-CELIV technique.

**Figure 4:**
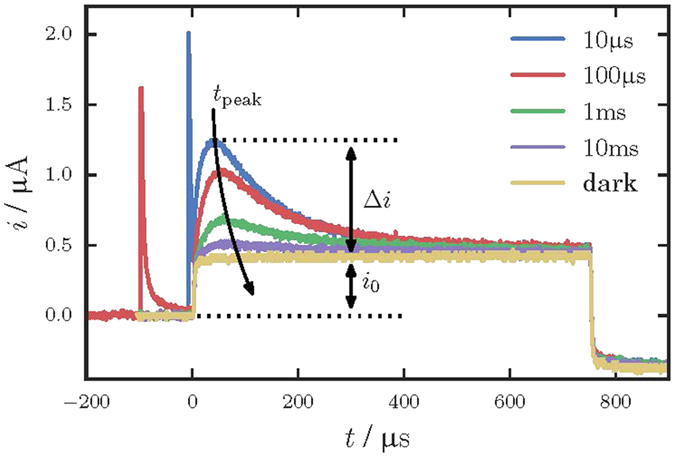
Dependence of the total current *i* and the photocurrent Δ*i* on the delay time *t*_*del*_ for the excitation with the uniformly absorbed light of 532 nm. The photocurrent Δ*i* decreases with time as carriers recombine.

Overall, the experiments were performed under conditions, which were shown to provide most accurate values of mobility^25^,^27^. Thus, uniformly absorbed light was used to generate carriers and the illumination level was adjusted to provide the magnitude of the photocurrent lower than the capacitive one. The measurements were repeated multiple times on different spots of the samples, providing essentially the same result. Therefore, the only major source of uncertainties in the mobility measurement is believed to come from how accurately the photo-peak position can be determined. The latter is limited by the width of the CELIV signal at the photo peak. As seen from Fig. 1b and Fig. 4, the peak position can be reasonably well defined within less than ±5% of the *t*_*peak*_ value. This results in relative uncertainty to be smaller than the symbol size in Figs 2 and 3.

### Experimental data on recombination of charge carriers

In order to investigate the recombination in PbO, measurements by the CELIV technique were performed at different light intensities and delay times *t*_*del*_. Fig. 4 shows the magnitude of the photocurrent Δ*i* as a function of the delay time at light intensity which allows Δ*i* to significantly exceed the magnitude of the capacitive signal *i*_0_.

The relatively low mobility value of carriers (*μ*_*h*_ ∼ 0.1 cm^2^/Vs at *F* = 1 V/μm) in poly-PbO suggests that recombination of carriers occurs in the so-called Langevin mode^32^,^33^. In the Langevin mode, the recombination rate is limited by the time, which carriers need to meet in space, i.e., recombination is limited by the transport process. Juska *et al*. performed a detailed analysis of the effect of recombination on the CELIV tracers, and showed that a photocurrent Δ*i* exceeding the magnitude of the capacitive signal *i*_0_ (like the one demonstrated in Fig. 4) unambiguously indicates that recombination is reduced in comparison to the conventional three-dimensional Langevin recombination^29^.

**Figure 5:**
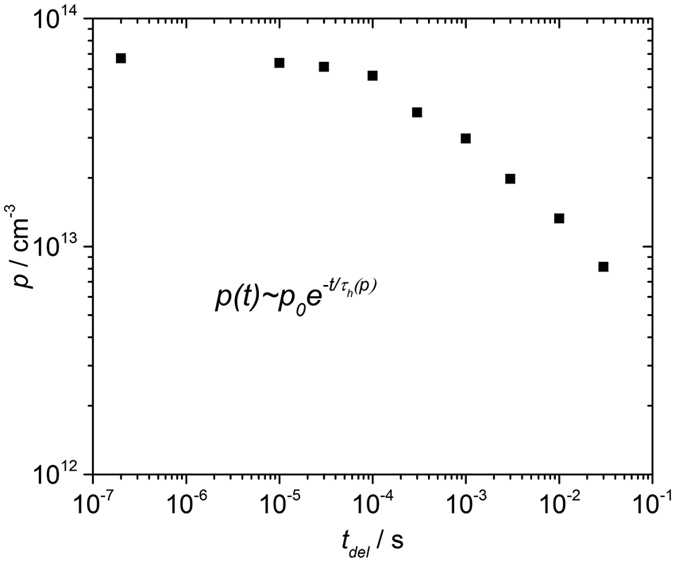
Photogenerated carrier density is plotted as a function of delay time *t*_*del*_.

Figure 4 also allows to estimate hole lifetime τ_*h*_ and mobility-lifetime product *μ*_*h*_*τ*_*h*_ for our experimental conditions. For this we calculated the density of the photogenerated holes *p* by integrating the area under the photocurrents shown in Fig. 4 and subtracting the contribution of the capacitive signal. The results are plotted in Fig. 5 as function of the delay time *t*_*del*_. As it is seen from Fig. 5, the carrier density remains constant for ∼30 μs. After this, *p* starts to decrease gradually, indicating the beginning of the recombination process. Taking into account the reduced Langevin recombination, we can assume that hole concentration depends on time as:


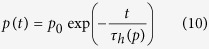


with the hole lifetime *τ*_*h*_(*p*) that depends on the carriers concentration *p*. The concentration-dependent carrier lifetime is inherent to any bimolecular recombination including the reduced Langevin one. Fitting our experimental results with the equation (10) in the vicinity of initial hole concentration yields *τ*_*h*_ ≈ 200 μs.

Taking *μ*_*h*_ ≈ 0.1 cm^2^/Vs at *F* = 1 V/μm, one can deduce that *μ*_*h*_*τ*_*h*_ = 2 × 10^−5^ cm^2^/V at the carrier concentration ∼5 × 10^13^ cm^−3^. It should be noted, that in the disordered materials with bimolecular type of recombination, lifetime depends on carrier concentration and mobility. In such, carrier lifetime should not be considered as a material parameter^34^. In contrast, *τ* is a characteristic of a device or experimental conditions^34^ and hence it is reported here in conjunction with the hole concentration *p*.

## Discussion

The hole mobility obtained from CELIV measurements depends strongly on the electric field and tend to reach ∼0.1 cm^2^/Vs at comparatively low *F* = 1 V/μm; electrons were found to be much slower carriers and did not contribute to the signal within the time scale of the performed measurements (see Fig. 1b). Remarkably, the hole mobility in PbO is similar to that in a-Se at room temperature, the only photoconductor, which is currently used commercially in direct conversion mammographic X-ray detectors. However, even though the carrier mobility values in these two materials are comparable, the transport mechanisms in a-Se and in PbO are of a fundamentally different nature. In a-Se, the hole mobility depends strongly on both the electric field *F* and the temperature *T*. Conduction in a-Se occurs in the frame of the so-called multiple trapping (MT) model, in which carriers move via extended states above the mobility edge, being trapped into and released from the localized states in the band tail of the valence band. Hole transport is dispersive at low temperatures only, and turns non-dispersive at room temperature^26^. The dispersion observed at low temperatures is due to the multiple trapping into energetically distributed trapping states^35^.

The most comprehensive and prominent mathematical description of the dispersive transport is based on the model of a continuous random walk of a charge carrier in a disordered material^36^. According to this picture, a carrier will be confronted with successively more and more difficult transitions in the course of its random motion, which would slow down the motion continuously, as described phenomenologically by equation (1). A microscopic transport mechanism behind this phenomenological treatment is to be clarified in each particular case depending on the experimentally observed features^32^. As has been shown by Pollak^37^, the broad dispersion of transit times requires traps, which catch charge carriers within times much shorter than those needed to release carriers out of these traps. This can be reached in a system with a sufficiently broad energy distribution of traps since carriers can be trapped rapidly from the transport states into energetically deep traps where they spend a long time before they are released back into transport states. Energy disorder has been identified in numerous organic (polymers) and inorganic (a-Si:H and a-Se) disordered materials as being responsible for the dispersive nature of charge transport. The energy distribution of traps in these materials leads to the broad distribution of the release times due to the exponential dependence of the release time on the trap energy^37^. As a result, mobility becomes temperature dependent.

In contrast, in the case of PbO, the hole mobility does not change significantly with *T*, while still depends strongly on *F* (see Figs 2 and 3). This temperature-independent behavior of the hole mobility rules out the MT as a possible transport mechanism in PbO. Indeed, if energy disorder would be responsible for charge transport providing, for instance, traps with energies below those corresponding to the transport path, rising *T* would enhance the release of carriers from the traps to the transport states and, concomitantly, it would increase the carrier mobility.

The same conclusion on the irrelevance of energy disorder for transport of holes in PbO can be reached by taking into account the observed independence of the dispersion parameter *α* on temperature *T*. If energy disorder is responsible for the dispersive nature of charge transport, the dispersion parameter α should be dependent on temperature^35^,^37^. Therefore, the evidenced independence of the dispersion parameter *α* on temperature in PbO in our study rules out energy disorder as a possible cause for the dispersive behavior of the hole mobility in this material.

In order to account for this temperature-independent dispersion parameter *α*, one should then search for mechanisms based on spatial disorder. In contrast to the assumption by Scher and Montroll^36^, hopping of carriers via randomly distributed isoenergetic sites would not lead to the broad dispersion of transient times at low electric fields, since carriers can always leave the traps as fast as they are captured^37^. At high electric fields the situation is changed because the high electric field prevents the release of carriers backwards with respect to the field direction and herewith restricts the random walk. If charge carriers cannot leave the areas, in which they are trapped by material inhomogenieties at the time scale of capture processes, the charge transport can become dispersive, with the dispersion parameter independent of *T*.

**Figure 6:**
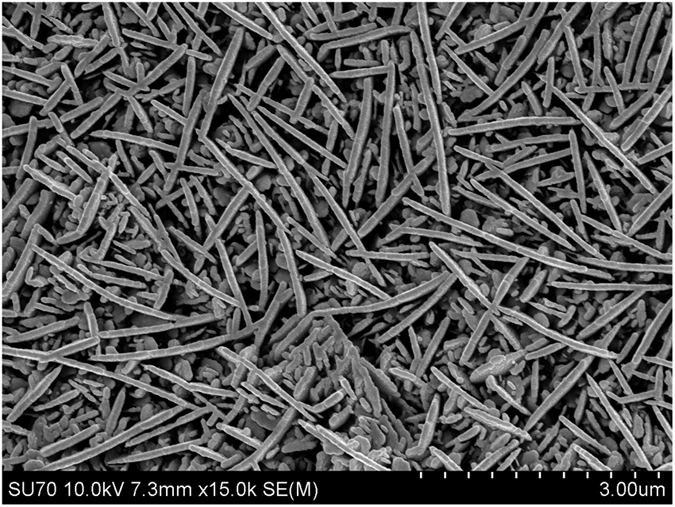
The SEM picture of the grown PbO layer.

Highly dispersive hole transport can be linked to the peculiarities of the polycrystalline structure of PbO layers. As it is seen in Fig. 6, the layer is a porous network of separate PbO platelets. Similar structure was previously repeatedly observed^17^,^18^,^21^,^22^,^38^ indicating a highly reproducible PbO thermal deposition process. It is believed that, at the mesoscopic level, each platelet consists of weakly coupled Pb-O layers characterized by highly anisotropic hole transport [ref. 39 and references therein]. At this point, we can only speculate that a very unusual structural configuration of poly-PbO provides spatial inhomogeneity, responsible for highly dispersive hole transport. The inhomogeneous structure of the material could also be responsible for the observed reduced carrier recombination, compared with that in spatially homogeneous systems.

We would like to mention, that due to the requirements of large area deposition all photoconductors that have been investigated for use in direct conversion medical imaging detectors, have disordered structures. In addition to a-Se and PbO, selected examples are: polycrystalline layers of PbI_2_^40^, HgI_2_^14^ and CdZnTe^41^. All of them exhibit dispersive transport that is typical for disordered materials. Regardless of its nature (whether this is energy or spatial disorder that causes dispersion), dispersive transport is trap-limited and is characterized by low drift mobilities. As a result, the drift mobility × lifetime product, *μτ*, can be insufficient, causing the distance drifted by the carrier before it is trapped or recombined (the so-called carrier *schubweg s* = *μτF*) to be shorter than the thickness of the photoconductive layer at practical electric fields. This results in incomplete charge collection, loss of detector sensitivity^42^,^43^,^44^,^45^, degradation of signal-to-noise ratio and image blur^19^,^23^. Trapped charge will be eventually collected, though this contributes to the undesired image lag and deteriorates temporal performance. The above problems, however, do not mean that low mobility materials with dispersive transport cannot be used in imaging. A plausible approach to achieve “detector grade” polycrystalline photoconductor is to improve a carrier schubweg, by increasing the applied electric field while reducing dark current. This, in turn, requires development of special blocking contacts similar to how this was done for a-Se^1^. In such an approach, the fundamental problem of low mobility dispersive transport can be bypassed. Once schubweg exceeds the layer thickness, the X-ray generated charge is successfully collected, thus making a material suitable for applications in X-ray imaging. As for the particular case of the polycrystalline PbO, the strong dependence of hole drift mobility on the applied field facilitates the task of improving schubweg since it increases with *F* rapidly.

Our findings suggest a new direction towards optimization of the PbO technology, which has previously been focused on the improvement of the layer stoichiometry to increase mobility and *μτ*. The latter approach may not be worth the effort since transport seems to be governed by spatial inhomogeneity of the PbO network, which is inherent to this material, rather than by a structure within a platelet.

## Methods

PbO layers were prepared from a high grade (5N) PbO powder using a thermal vacuum evaporation technique as reported elsewhere^17^,^18^. The deposition takes place in the atmosphere of molecular oxygen at the partial pressure of ∼4 × 10^−3^ mbar. The PbO vapor condenses on ITO covered glasses in a form of platelets, which are several microns in diameter and about a hundred nanometers thick. The scanning electron micrograph of the grown layer is shown in Fig. 6. For mobility measurements, a metal contact (Au) 2 mm in diameter is deposited *ex-situ* on the PbO surface in a different vacuum chamber. All experiments were performed on PbO layers with the thickness *d* = 5 μm.

The hole drift mobility, transport mechanism and recombination were characterized by conventional time-of-flight (TOF) and photo-CELIV measurements. TOF is known to be one of the major tools for direct determination of the charge transport mechanism^24^. In this technique charge carriers are generated by strongly absorbed light and their drift through the sample is caused by the external constant electric field. Since carriers are generated close to a sample surface, polarity of the applied bias governs the type of moving carriers. Carrier transit time, *t*_*tr*_, is determined from the characteristic kink of the quasi-rectangular current transients. However, in non-crystalline materials with dispersive carrier transport, the shape of the current transient often deviates significantly from the ideal rectangular shape, making it difficult to measure carrier transit times.

**Figure 7:**
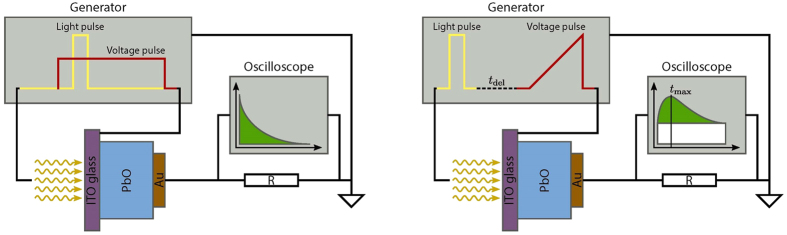
Schematic representation of the TOF (**a**) and photo-CELIV (**b**) apparatuses. The voltage from the generator was applied to the sample through the ITO contact. The signal was read out from the top (Au) contact and observed as a voltage drop on the oscilloscope resistance *R*.

Charge extraction by linear increasing voltage (photo-CELIV) has been proposed to overcome the problems associated with TOF measurements^25^. In photo-CELIV, charge carriers are photo-generated in unbiased sample. After a preset delay time, a linearly increasing voltage ramp is applied to the sample in order to extract the photo-generated charge. As voltage increases, more carriers are extracted and the photocurrent rises. Once most of the carriers have been collected, the photocurrent decays. Mobility is derived from the time (*t*_*peak*_) needed to reach the peak of the photocurrent. The typical TOF and photo-CELIV experimental apparatuses are shown in Fig. 7a,b, respectively. For the TOF measurements, the charge carriers were generated by 35 ps laser pulses with the wavelength of 355 nm, while a constant bias was applied to the sample. The sample was irradiated from the ITO side, to which positive polarity was applied. For the photo-CELIV measurements, excitation wavelengths of 355 nm and 532 nm were used. For 355 nm, the attenuation depth in PbO is ∼0.2 μm (i.e., light is absorbed close to the sample surface), while for 532 nm, the attenuation depth is ∼17 μm^38^, which provides uniform absorption for the sample thickness *d* = 5 μm. In order to investigate the temperature dependencies of the carrier mobility, the sample was placed in a cooling-heating stage, which allowed precise temperature control over a wide temperature range.

## Additional Information

**How to cite this article**: Semeniuk, O. *et al*. Charge transport mechanism in lead oxide revealed by CELIV technique. *Sci. Rep*. **6**, 33359; doi: 10.1038/srep33359 (2016).
